# A High-Accuracy Model Based on Plasma miRNAs Diagnoses Intrahepatic Cholangiocarcinoma: A Single Center with 1001 Samples

**DOI:** 10.3390/diagnostics11040610

**Published:** 2021-03-29

**Authors:** Jie Hu, Yi-Ning Wang, Dan-Jun Song, Jin-Peng Tan, Ya Cao, Jia Fan, Zheng Wang, Jian Zhou

**Affiliations:** 1 Liver Surgery Department, Liver Cancer Institute, Zhongshan Hospital, Fudan University, Shanghai 200032, China; hu.jie3@zs-hospital.sh.cn (J.H.); yiningwang19@fudan.edu.cn (Y.-N.W.); songdanjun@hotmail.com (D.-J.S.); 20211210060@fudan.edu.cn (J.-P.T.); jiafan99@yahoo.com (J.F.); 2 Key Laboratory of Carcinogenesis and Cancer Invasion, Ministry of Education, Shanghai 200032, China; 3 Cancer Research Institute, Xiangya School of Medicine, Central South University, Changsha 410078, China; ycao98@csu.edu.cn; 4 Institute of Biomedical Sciences, Fudan University, Shanghai 200032, China

**Keywords:** intrahepatic cholangiocarcinoma, CA19-9, microRNA, diagnosis, circulating biomarker

## Abstract

Objectives: Intrahepatic cholangiocarcinoma (iCCA) is a highly malignant cancer. More than 70% of patients are diagnosed at an advanced stage. The aim of this study was to evaluate the diagnostic value of plasma miR-21, miR-122, and CA19-9, hoping to establish a novel model to improve the accuracy for diagnosing iCCA. Materials and methods: Plasma miR-21 and miR-122 were detected in 359 iCCA patients and 642 controls (healthy, benign liver lesions, other malignant liver tumors). All 1001 samples were allocated to training cohort (*n* = 668) and validation cohort (*n* = 333) in a chronological order. A logistic regression model was applied to combine these markers. Area under the receiver operating characteristic curve (AUC) was used as an accuracy index to evaluate the diagnostic performance. Results: Plasma miR-21 and miR-122 were significantly higher in iCCA patients than those in controls. Higher plasma miR-21 level was significantly correlated with larger tumor size (*p* = 0.030). A three-marker model was constructed by using miR-21, miR-122 and CA19-9, which showed an AUC of 0.853 (95% CI: 0.824–0.879; sensitivity: 73.0%, specificity: 87.4%) to differentiate iCCA from controls. These results were subsequently confirmed in the validation cohort with an AUC of 0.866 (0.825–0.901). The results were similar for diagnosing early (stages 0–I) iCCA patients (AUC: 0.848) and CA19-9^negative^ iCCA patients (AUC: 0.795). Conclusions: We established a novel three-marker model with a high accuracy based on a large number of participants to differentiate iCCA from controls. This model showed a great clinical value especially for the diagnosis of early iCCA and CA19-9^negative^ iCCA.

## 1. Introduction

Intrahepatic cholangiocarcinoma (iCCA) is a rare, highly malignant and fatal primary epithelial cancer arising above the second-order bile ducts [1]. iCCA is the second most common primary hepatic malignancy after hepatocellular carcinoma (HCC), comprising approximately 15% of all primary liver cancers and 3% of gastrointestinal cancers [2]. In contrast to HCC, iCCA usually develops in non-cirrhotic liver which makes it hard for surveillance. iCCAs have insidious onset without specific symptoms, therefore most patients are diagnosed at an advanced stage with multiple lesions within the liver, lymph node, and/or distant metastasis, missing the opportunity for curative resection. The etiology of iCCA has not been clearly illustrated yet, and most cases occurred sporadically without recognizable risk factors.

The rising incidence and mortality rate of iCCA [3] attracted growing interests among clinicians and scientists. Despite recent progress in the recognition, diagnosis, and therapies for iCCA [1,4,5], the prognosis of this gloomy cancer remains dismal. Most iCCA patients occur in the absence of known risk factors, thus the only opportunity for early diagnosis is by cross-sectional imaging carried for other reasons [1,6]. Incapacity to detect iCCA early leads to the advanced staging at the time of diagnosis, which is one of the main reasons for poor prognosis in iCCA patients (only 20–30% iCCA patients are candidates for curative intent surgery [5,7]). Screening and validation of effective biomarkers in circulating biofluids (serum/plasma) may change the paradigm in disease diagnosis and management. MicroRNAs (miRNAs) are a group of small noncoding RNAs, regulating gene expression at the post-transcriptional level and promote cancer development and metastasis. Circulating miRNAs detected in biofluids after active secretion or releasing from dying cells are promisingly early diagnostic tools for various kinds of cancers. Due to the etiological complexity and heterogeneity of iCCA, a generally acknowledged miRNA profile with high diagnostic efficacy has not been established for iCCA yet. Among miRNAs reported in previous studies, miR-21 and miR-122 are two commonly recognized cancer-associated miRNAs in the carcinogenesis of iCCA, which also demonstrate the potential of iCCA diagnostic biomarkers. Here we evaluated the diagnostic value of plasma miR-21 and miR-122 in 1001 samples (including 359 iCCA patients and 642 controls) and compared them with the traditional marker, carbohydrate antigen 19-9 (CA19-9), We found iCCA patients showed higher expression levels of the plasma miR-21 and miR-122 and we constructed a logistic regression model (combining miR-21, miR-122, and CA19-9) which showed a high diagnostic power for iCCA.

## 2. Patients and Methods

### 2.1. Study Design and Patients

**Figure 1:**
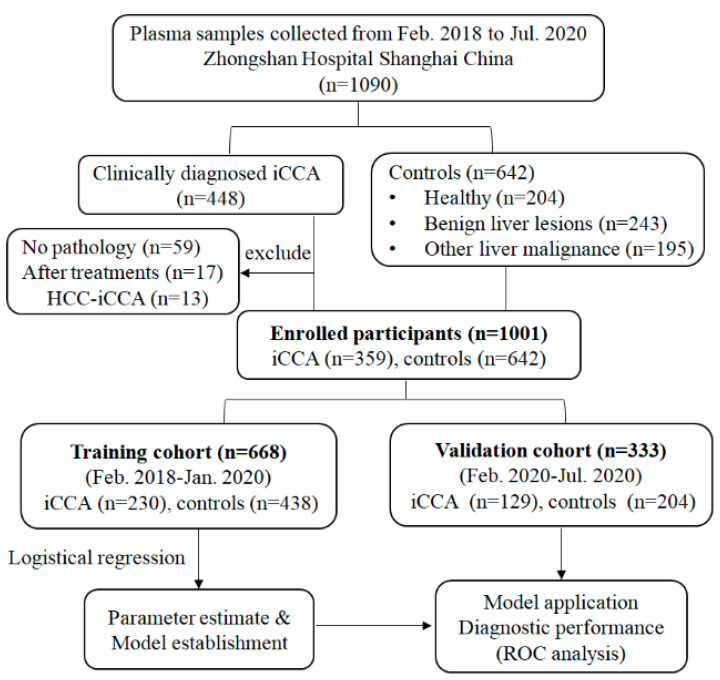
Flow chart of study design. iCCA, intrahepatic cholangiocarcinoma; HCC-iCCA, combined hepatocellular carcinoma and intrahepatic cholangiocarcinoma; ROC, receiver operating characteristics.

Plasma samples were obtained from iCCA patients who received surgical treatment or liver biopsy in Zhongshan hospital, Fudan University between February 2018 and July 2020. The diagnosis was determined by two experienced pathologists and required to meet the following criteria: (a) The diagnosis of iCCA was pathologically confirmed, mixed liver cancer with iCCA and HCC were excluded. (b) None of the patients had received anti-cancer treatment previously. (c) No history of other malignancies. The clinicopathological features of iCCA patients including age, gender, treatment history, laboratory tests, pathological diagnosis, tumor number and size, tumor differentiation, lymph node metastasis, microvascular invasion (MVI) were recorded. Control group including healthy, benign liver lesions (Focal Nodular Hyperplasia (FNH), Angiomyolipoma (AML), Cyst, Adenoma) and other malignant diseases of liver (HCC, Colorectal Liver Metastasis (CRLM), other secondary liver cancer). This study was approved by the Ethical Committee of the Zhongshan hospital, Fudan University and performed in accordance with Declaration of Helsinki. Written informed consent was obtained from each patient prior to the study. All samples were allocated to two phases in chronological order (Figure 1).

### 2.2. Quantification of Plasma miR-21 and miR-122

Plasma samples were extracted within four hours of venipuncture. All the samples were first processed by a two-step centrifugation method: first spun at 1300 *g* for 20 min to remove the majority of blood cells and a second spin at 14,000 *g* for another 10 min to remove the cellular debris. The plasma was diluted with preservative fluid (JUSBIO SCIENCES, Shanghai, China, FD05059). miR-21 and miR-122 were extracted and quantified by plasma miRNA testing kit (JUSBIO SCIENCES, Shanghai, China, HCC9655) according to the manufacturer’s protocol. The expression level of miR-1228 was used as a stable endogenous control for normalization [8]. All assays were carried out in triplicate.

### 2.3. Model Construction and Validation

All the 1001 samples that met the eligibility criteria were allocated to two phases (2:1) in chronological order. The training cohort (*n* = 668) was selected to construct the diagnostic panel based on the logistic regression model for the differentiation between the iCCA patients and the control group. The validation cohort (*n* = 333) was adopted for independent validation of the model.

### 2.4. Statistical Analysis

All the results were represented as mean ± standard error unless specific indication. Comparison between two groups was performed with unpaired Student’s *t*-test, ANOVA or Bonferroni’s tests were used for multiple comparisons when appropriate. A logistic regression model was used to combine these markers based on the training dataset. The predicted probability of being diagnosed with iCCA was used as a surrogate marker to construct receiver operating characteristic (ROC) curve. Area under the ROC curve (AUC) was used as an accuracy index for evaluating the diagnostic performance of miR-21, miR-122, CA19-9 or the combined markers. All statistical analyses were performed by using GraphPad Prism 8.0 software (San Diego, CA, USA), JMP software (version 9.02; SAS Institute, Cary, NC, USA), MedCalc software (version 10.4.7.0; Mariakerke, Belgium), and R software (version 3.4.1, Vienna, Austria). A *p*-value < 0.05 was regarded as statistically significant.

### 2.5. Target Gene Prediction and Enrichment Analysis

Target genes of miR-21, miR-122 were predicted by using Tarbase v.7.0 in DIANA miRPath v.3 (http://www.microrna.gr/miRPathv3 accessed on 22 January 2021). Enrichment analysis of target genes was performed by Kyoto Encyclopedia of Genes and Genomes (KEGG). Significant enrichment results were defined as *p*-value < 0.05.

## 3. Results

### 3.1. Patient Characteristics

The characteristics of the study participants were presented in Table S1. There was no significant difference in the distribution of age, sex and the composition of diseases in controls between the training and validation cohorts. There were totally 448 patients clinically diagnosed as iCCA, while 50 patients excluded for no pathologic diagnosis, 17 patients excluded because of anti-cancer treatment history (chemotherapy and/or transhepatic arterial chemotherapy and embolization). Thirteen patients with mixed cancer cell of HCC and iCCA were also excluded. Finally, 359 iCCA patients were enrolled in this study (Table 1). Among them, 266 patients received radical surgery while 93 patients with incurable disease received the pathologic diagnosis through liver biopsy. The median CA19-9 level in iCCA with radical surgery was 40.4 IU/mL comparing to 339.0 IU/mL in iCCA received biopsy. The tumor sizes were 4.87 (±2.78) cm and 7.09 (±2.90) cm in iCCA with radical surgery and in iCCA with biopsy, respectively. For iCCA patients receiving surgery, 18.8% (50/266) have multiple tumors, 67.3% (179/266) tumors infiltrated the hepatic capsule, 28% (75/266) showed microvascular invasion, while 15.8% (42/266) had local lymphatic metastasis.

We enrolled 642 control participants from three kinds of population (Table S1), including healthy individual (*n* = 204), patients with benign liver lesions (45 FNH, 54 hemangioma, 45 cyst, 13 AML, 19 adenoma, and 51 other benign lesions), other malignant diseases of liver (12 HCC, 124 CRLM, 28 other secondary liver cancers, and 31 other malignant lesions). All controls with liver lesions received pathologic diagnosis. All healthy controls received abdominal ultrasonography to confirm the absence of liver space-occupying lesions.

### 3.2. The Expression of Plasma miR-21 and miR-122

**Figure 2:**
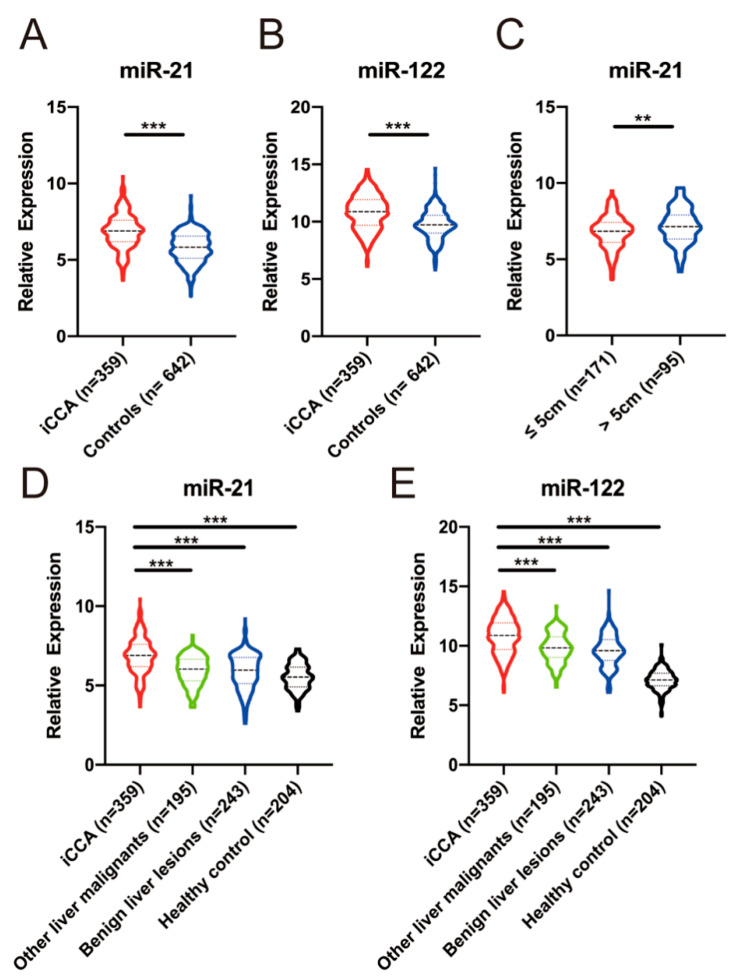
Relative expression of plasma miR-21 and miR-122 in iCCA and control patients. (**A**,**B**), The relative expression of plasma miR-21 and miR-122 in iCCA patients was significantly higher than that in control (*p* < 0.001). (**C**) The relative expression of plasma miR-21 in iCCA patients with > 5cm tumor size was significantly higher than iCCA patients with ≤ 5 cm tumor size (*p* = 0.009). (**D**,**E**), The relative expression of plasma miR-21 and miR-122 in iCCA patients was significantly higher than that in healthy, benign liver lesions, and other liver malignancies (all *p* < 0.001). **, *p* < 0.01; ***, *p* < 0.001.

Plasma miR-21 and miR-122 were detected by using qRT-PCR. The expression of plasma miR-21 and miR-122 in iCCA group were significantly higher than in control group (*p* < 0.001 and *p* < 0.001 respectively, Figure 2). The expression levels of miR-21 and miR-122 decreased in turn in iCCA, other liver malignancies, benign liver lesions, and healthy controls and the expression level in iCCA patients was higher than in other three control group respectively (Figure 2).

The clinical associations between the expression of plasma miR-21/miR-122 and clinicopathological characteristics in iCCA patients receiving radical surgery was evaluated (Table 2). We found higher plasma miR-21 level was significantly correlated to larger tumor size (*p* = 0.030), and iCCA patients with > 5 cm tumor size showed higher plasma miR-21 level (*p* = 0.009, Figure 2C). While the expression of plasma miR-122 showed no correlation with any listed clinicopathological characteristics.

### 3.3. Model Construction in Training Cohort

**Figure 3:**
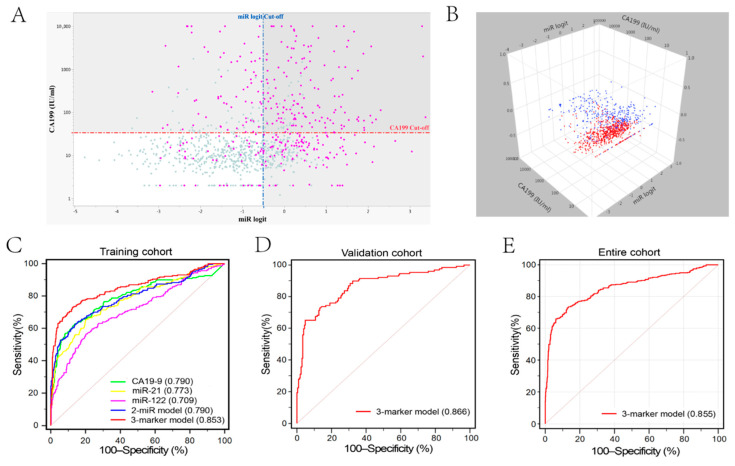
Diagnostic performance of CA19-9, miR-21, miR-122, 2-miR model and 3-marker model for iCCA. (**A**,**B**) The demonstration of CA19-9 level and 2-miR model in entire cohort by using two-dimension scatterplot and three-dimension scatterplot. iCCA patients (87.7%, 315/359) could be diagnosed by a combination of CA19-9 level and 2-miR model (gray shadow region). Red dotted line, cut-off value for CA19-9 (34 IU/mL); blue dotted line, cut-off value for 2-miR model (−0.494). (**C**) Diagnostic performance of five parameters for iCCA diagnosis in the training cohort. The AUC of three-marker model was significantly larger than CA19-9 (*p* = 0.009), miR-21 (*p* < 0.001), miR-122 (*p* < 0.001), and 2-miR model (*p* < 0.001). (**D**,**E**) Diagnostic performance of three-marker model in the validation cohort and entire cohort.

High expression levels of miR-21 (Fold change = 1.21, *p* < 0.001) and miR-122 (Fold change = 1.12, *p* < 0.001) were observed in iCCA patients compared with those in the control group. The diagnostic accuracy (AUC) of miR-21, miR-122 and CA19-9 was 0.773, 0.709 and 0.790, respectively (Table 3). We combined miR-21 and miR-122 by using logistic regression model: logit (*p* = iCCA) = −9.289 + (0.793 × miR-21) + (0.353 × miR-122). However, miR-122 showed lower diagnostic accuracy than CA19-9 (*p* = 0.005) but comparable accuracy to miR-21 (*p* = 0.557) or two-miR model (*p* = 0.999). When combining the two-miR model and CA19-9 together, we found 87.7% (315/359) of iCCA patients could be screened out from the controls (Figure 3A,B). Therefore, in order to establish a diagnostic model with higher accuracy, we combined these three circulating markers (miR-21, miR-122, and CA19-9) by using a logistic regression model in the training cohort of 668 samples. The predicted probability of diagnosing iCCA based on the logit model [logit (*p* = iCCA) = −9.967 + (0.777 × miR-21) + (0.389 × miR-122) + (0.004 × CA19-9)] was used to construct the ROC curve. The diagnostic performance of the three-marker panel with AUC of 0.853 (95% CI, 0.824–0.879; Sensitivity = 73.0%, Specificity = 87.4%, Figure 3) was significantly higher than that of each single marker or two-miRNA panel (all *p* < 0.05).

### 3.4. Model Validation

The three-marker model established from the training cohort was used to predict the probability of iCCA diagnosis in the independent validation cohort with 333 participants. We found that the diagnostic accuracy of the three-marker model in validation cohort (AUC = 0.866, 95% CI, 0.825–0.901; sensitivity = 65.1%, specificity = 95.1%) or the entire cohort (AUC = 0.855, 95% CI, 0.832–0.876; sensitivity = 73.0%, specificity = 87.1%, Table S2) was comparable to that in the training cohort.

**Figure 4:**
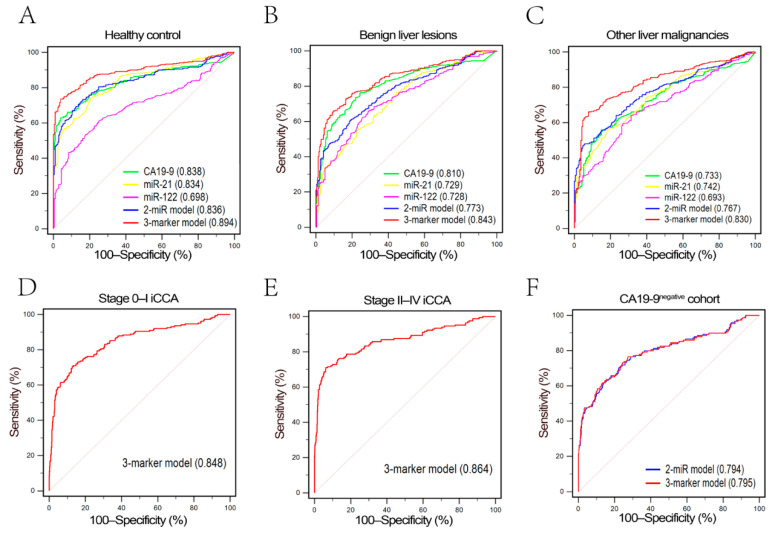
Subgroup ROC analysis for iCCA diagnosis. (**A**–**C**) Diagnostic performance of five parameters in healthy control subgroup, benign liver lesions subgroup and other liver malignancies subgroup. Three-marker model also demonstrated a preferable diagnostic efficacy than other parameters in three subgroups (Tables S3–S5). (**D**,**E**) Diagnostic efficacy of three-marker model for iCCA with different AJCC stage. Three-marker model still possessed a good diagnostic efficacy even in early stage iCCA. (**F**) Diagnostic efficacy of two-miR model and three-marker model for iCCA in CA19-9^negative^ cohort.

The performance of three-marker model in differentiating iCCA from the healthy, benign liver lesions and other malignant liver lesions was also evaluated, respectively (Tables S3–S5). Three-marker model had a high accuracy in discriminating iCCA from healthy, benign liver lesions, and other malignant liver lesions (AUC was 0.894, 0.843, and 0.830, respectively, Figure 4A–C). When focusing the subgroups of healthy and benign liver lesions participants, we found each single marker also showed relatively high diagnostic values (Tables S3–S5). The AUCs of CA19-9 in differentiating iCCA from the healthy and benign liver lesions were 0.838 and 0.810, respectively, while the AUCs of miR-21 in differentiating iCCA from the healthy and benign liver lesions participants were 0.834 and 0.729, respectively.

We further evaluated the diagnostic performance of the three-marker model in different AJCC stages (Table S6). The AUCs for iCCA patients with AJCC stages 0–I and II–IV were 0.848 (0.821–0.871) and 0.864 (0.821–0.871), respectively. These results demonstrated that the diagnostic value of the three-marker model was independent of the disease stage. We also tested the diagnostic value of the model in CA19-9^negative^ participants. The diagnostic performance of the three-marker model (AUC = 0.795, 95% CI, 0.763–0.824) for CA19-9^negative^ iCCA patients was similar to two-miR model (AUC = 0.794, 95% CI, 0.762–0.823, Table 4).

### 3.5. Function Prediction of miR21 and miR-122

Using Tarbase v.7.0, we got 2181 and 1548 potential target genes for miR-21 and miR-122 respectively. KEGG pathway analysis revealed that the target genes of miR-21 were extensively involved in iCCA-associated signaling pathways (the MAPK [9], FoxO [10], and p53 [11,12] signaling pathway), suggesting their potential roles in iCCA pathogenesis (Figure S1). While the target genes of miR-122 were involved in the PI3K-AKT [13], AMPK [14], TGF-β [15,16] signaling pathway.

## 4. Discussion

iCCA is an aggressive primary liver cancer with an extremely poor prognosis (The long-term survival for iCCA is even worse than for HCC). In contrast to HCC, we know less about the epidemiology and etiology of iCCA. Inflammation and subsequent injury to the bile ducts may play a role in the carcinogenesis of iCCA [17]. The increasing incidence of obesity and NASH (non-alcoholic steatohepatitis) may account for the rising occurrence of cholangiocarcinoma, particularly in the western countries [18,19]. CA19-9 is a traditional biomarker for cholangiocarcinoma. However, its sensitivity (42.7–72%) for the diagnosis of iCCA is not satisfactory [20]. A lack of effective early diagnostic strategy and the asymptomatic nature of iCCA consequently lead to patients presenting with late-stage disease not amenable to curative treatment. To improve diagnosis, prognosis, and monitor therapeutic response, novel biomarkers are urgently warranted [21].

MiRNAs in bodily fluids are promising minimally invasive biomarkers for tumor diagnosis, progression monitoring, and therapeutic response prediction [22,23]. In this study, we found the diagnostic power of each single marker was not satisfied (the AUC was around 0.750), which may be attributed to the tumor heterogeneity. Cancers are the cumulative results of polygenic alterations and their interactions and single molecular marker is unable to representative of the entire iCCA population. As such, we constructed a logistic regression model (combining miR-21, miR-122, and CA19-9) which showed a higher diagnostic power for iCCA. Most importantly, this three-marker model demonstrated a high accuracy in the diagnosis of early iCCA (AJCC stage 0–I), which made it qualified the potential for early diagnosis and screening. Currently, CA19-9^negative^ iCCA patients lack tumor marker for disease diagnosis and treatment monitoring. Our model showed a high diagnostic value even for CA19-9^negative^ iCCA patients, which suggested that plasma miR-21 and miR-122 were the important complementation of CA19-9 in the monitoring of iCCA.

MiR-21 is well recognized as an oncomir, which is highly expressed in cholangiocarcinoma tissue compared with the noncancerous biliary epithelium [24,25,26,27]. It plays important roles in the progress of cholangiocarcinoma through targeting PTEN/AKT pathway [27,28], TIMP3 (tissue inhibitor of metalloproteinases 3) [29], 15-PGDH (15-hydroxyprostaglandin dehydrogenase) [24], or EMT (epithelial–mesenchymal transition) [30]. We found that higher plasma miR-21 level was significantly associated with larger tumor size, implying a close correlation between miR-21 and tumor proliferation. While miR-122, which downregulated in tumor tissues of iCCA patients [31], is considered a tumor suppressor miRNA for iCCA [32,33]. Interestingly, miR-122 was found highly expressed in plasma of iCCA patients comparing to controls [34]. This result was also confirmed by our study. This phenomenon may be ascribed to the fact that miR-122 is the most abundant miRNA in the liver [35], persistent chronic inflammation and subsequent injuries to the liver in iCCA patients lead to a release of miR-122 to the circulation.

There were a few studies attempting to evaluate circulating miRNAs as possible blood-based biomarkers for noninvasive diagnosis of cholangiocarcinoma. Kishimoto et al. and Correa-Gallego et al. reported plasma miR-21 as a diagnostic biomarker for cholangiocarcinoma patients [26,36]. However, their studies were limited by small sample size (*n* = 94 and *n* = 25 respectively) and control types (50 healthy 23 benign biliary disease [36] and 7 healthy [26] respectively). Circulating miR-26a [37], miR-122 [38,39], miR-150 [40], miR-483-5p, and miR-194 [38] also have been reported as biomarkers for cholangiocarcinoma patients. However, the limitation of small sample size, missing independent validation, absence of stable internal control for the quantification of miRNAs, and require different miRNAs to discriminate different control groups raised concern about the robustness of the miRNA markers. The reported AUCs in previous studies for diagnosing iCCA vary greatly from 0.7 to 0.9, which was ascribed to the selection bias of controls. In our results, the diagnostic power was also higher when only healthy individuals were selected as controls in the subgroup analysis (0.838 and 0.834 for CA19-9 and miR-21 respectively). However, in clinical circumstance, we need to differentiate patients from far more complicated background. An excellent marker is required to have the ability to screen target disease from people at high risk of getting this disease or from people with other similar diseases.

In summary, we established a novel model combining plasma miRNAs and CA19-9 in a large number of participants that differentiates iCCA from controls with high accuracy. This model showed great clinical value for the screening of early iCCA and CA19-9^negative^ iCCA. To our knowledge, this is the largest sample set ever reported regarding plasma miRNA and iCCA. However, the present study had several limitations. Our study was conducted in single hospital and the model was established and validated based on Chinese population. The results still need to be further verified by multi-center clinical trials with larger sample sizes from different race.

## Figures and Tables

**Table 1:** Baseline characteristics and clinicopathological characteristics in iCCA patients

Characteristics	iCCA Cohort		
	Total iCCA (*n* = 359)	Radical Surgery (*n* = 266)	Liver Biopsy (*n* = 93)
Age > 60 yrs	215 (59.9%)	158 (59.4%)	57 (61.3%)
Male	218 (60.7)	160 (60.2%)	58 (62.4%)
Diagnosed by specimen after			
Radical surgery	266 (74.1%)	266 (100.0%)	−
Liver resection	261 (72.7%)	261 (98.1%)	−
Liver transplantation	5 (1.4%)	5 (1.9%)	−
Liver biopsy	93 (25.9%)	−	93 (100.0%)
Serum tumor biomarker, median (range)			
CA19-9 (IU/mL)	57.2 (2.0–10,000.0)	40.4 (2.0–10,000.0)	339.0 (2.0–10,000.0)
AFP (ng/mL)	2.9 (0.9–19,998)	2.9 (0.9–19,998)	3.0 (0.9–1115.0)
Tumor characteristics			
Tumor size (cm)	5.44 (±2.96)	4.87 (±2.78)	7.09 (±2.90)
Multiple	73 (20.3%)	50 (18.8%)	23 (24.7%)
Capsular invasion	−	179 (67.3%)	−
Perineural invasion	−	69 (25.9%)	−
MVI	−	75 (28.2%)	−
AJCC Stage II–IV	169 (47.1%)	93 (35.0%)	76 (81.7%)
Lymphatic metastasis	−	42 (15.8%)	−

iCCA, intrahepatic cholangiocarcinoma; CA19-9, Carbohydrate antigen19-9; AFP, alpha fetoprotein; AJCC, American Joint Committee on Cancer; MVI, Microvascular Invasion.

**Table 2:** Clinical associations between the expressions of plasma miR-21/miR-122 and clinicopathological characteristics of iCCA patients received radical surgery

Characteristics	iCCA Patients Receiving Radical Surgery (*n* = 266)					
	Plasma miR-21			Plasma miR-122		
	Low (*n* = 133)	High (*n* = 133)	*p*	Low (*n* = 133)	High (*n* = 133)	*p*
Tumor size, cm			0.030			0.096
≤5 cm	94	77		79	92	
>5 cm	39	56		54	41	
Tumor number			0.754			1.000
Single	107	109		108	108	
Multiple	26	24		25	25	
Capsular invasion						
No	39	48		36	51	
Yes	94	85		97	82	
Microvascular invasion			0.496			0.683
No	98	93		97	94	
Yes	35	40		36	39	
Perineural invasion						
No	98	99		101	96	
Yes	35	34		32	37	
Lymphatic metastasis						
No	112	112		107	117	
Yes	21	21		26	16	
CA19-9 level, IU/mL						
≤ 34	63	63		64	62	
> 34	70	70		69	71	
AJCC Stage			0.898			0.247
0−I	87	86		82	91	
II−III	46	47		51	42	

The low and high expression of miR-21 and miR-122 was defined by using median as the cut-off.

**Table 3:** Diagnostic performance of circulating markers for iCCA in the training and validation cohorts.

Markers	iCCA vs. Control											
	Training Cohort						Validation Cohort					
	AUC (95% CI)	NPV (%)	PPV (%)	Sensitivity (%)	Specificity (%)	*p*	AUC (95% CI)	NPV (%)	PPV (%)	Sensitivity (%)	Specificity (%)	*p*
CA19-9	0.790 (0.757–0.820)	72.1	87.7	62.6	86.3	<0.001	0.805 (0.759–0.846)	68.8	86.7	62.8	86.8	<0.001
miR-21	0.773 (0.740–0.805)	77.3	71.0	65.7	79.7	<0.001	0.785 (0.737–0.828)	72.5	65.0	72.9	70.6	<0.001
miR-122	0.709 (0.673–0.744)	73.7	65.5	56.5	70.7	<0.001	0.707 (0.655–0.755)	71.6	69.9	53.5	80.9	<0.001
2-miR model *	0.790 (0.757–0.820)	80.1	73.9	63.9	84.7	<0.001	0.801 (0.754–0.842)	75.8	69.8	81.4	69.6	<0.001
3-marker model ^#^	0.853 (0.824–0.879)	83.3	86.5	73.0	87.4	<0.001	0.866 (0.825–0.901)	81.0	87.5	65.1	95.1	<0.001

* Logit (*p* = iCCA) = −9.289 + (0.793 × miR-21) + (0.353 × miR-122); ^#^ Logit (*p* = iCCA) = −9.967 + (0.777 × miR-21) + (0.389 × miR-122) + (0.004 × CA19-9); AUC, Area Under Curve; CI, confidence interval; NPV, negative predictive value; PPV, positive predictive value.

**Table 4:** Diagnostic performance of circulating markers for iCCA patients with negative CA19-9.

Markers	iCCA Cohort with Negative CA19-9 (*n* = 149) vs. Control (*n* = 571)					
	AUC (95% CI)	NPV (%)	PPV (%)	Sensitivity (%)	Specificity (%)	*p*
miR-21	0.771 (0.739–0.802)	83.4	80.4	77.2	65.8	<0.001
miR-122	0.712 (0.677–0.725)	81.8	82.1	63.8	74.1	<0.001
2-miR model *	0.794 (0.762–0.823)	85.5	84.4	70.5	77.6	<0.001
3-marker model ^#^	0.795 (0.763–0.824)	85.9	85.1	76.5	72.7	<0.001

* Logit (*p* = iCCA) = −9.289 + (0.793 × miR-21) + (0.353 × miR-122); ^#^ Logit (*p* = iCCA) = −9.967 + (0.777 × miR-21) + (0.389 × miR-122) + (0.004 × CA19-9); AUC, Area Under Curve; CI, confidence interval; NPV, negative predictive value; PPV, positive predictive value.

## Data Availability

The data presented in this study are available on request from the corresponding author. The data are not publicly available due to ethical restrictions.

## Supplementary Materials

The following are available online at https://www.mdpi.com/article/10.3390/diagnostics11040610/s1, Figure S1: Enrichment analysis for predicted target genes of miR-21 and miR-122; Table S1: Baseline characteristics of included participants in the training and validation cohort; Table S2: The diagnostic performance of circulating markers for iCCA in the entire cohort; Tables S3–S5: The diagnostic performance of circulating markers between iCCA and subgroup control; Table S6: The diagnostic performance of circulating markers for iCCA with different AJCC stage.
