# Supplementary material for: A High-Accuracy Model Based on Plasma miRNAs Diagnoses Intrahepatic Cholangiocarcinoma: A Single Center with 1001 Samples
Source: Diagnostics (Basel). 2021 Mar 29;11(4):610. doi: 10.3390/diagnostics11040610 (PMC8066692; doi:10.3390/diagnostics11040610)
Supplement: Supplementary file 1 [file diagnostics-11-00610-s001.pdf]

Supplementary Figure

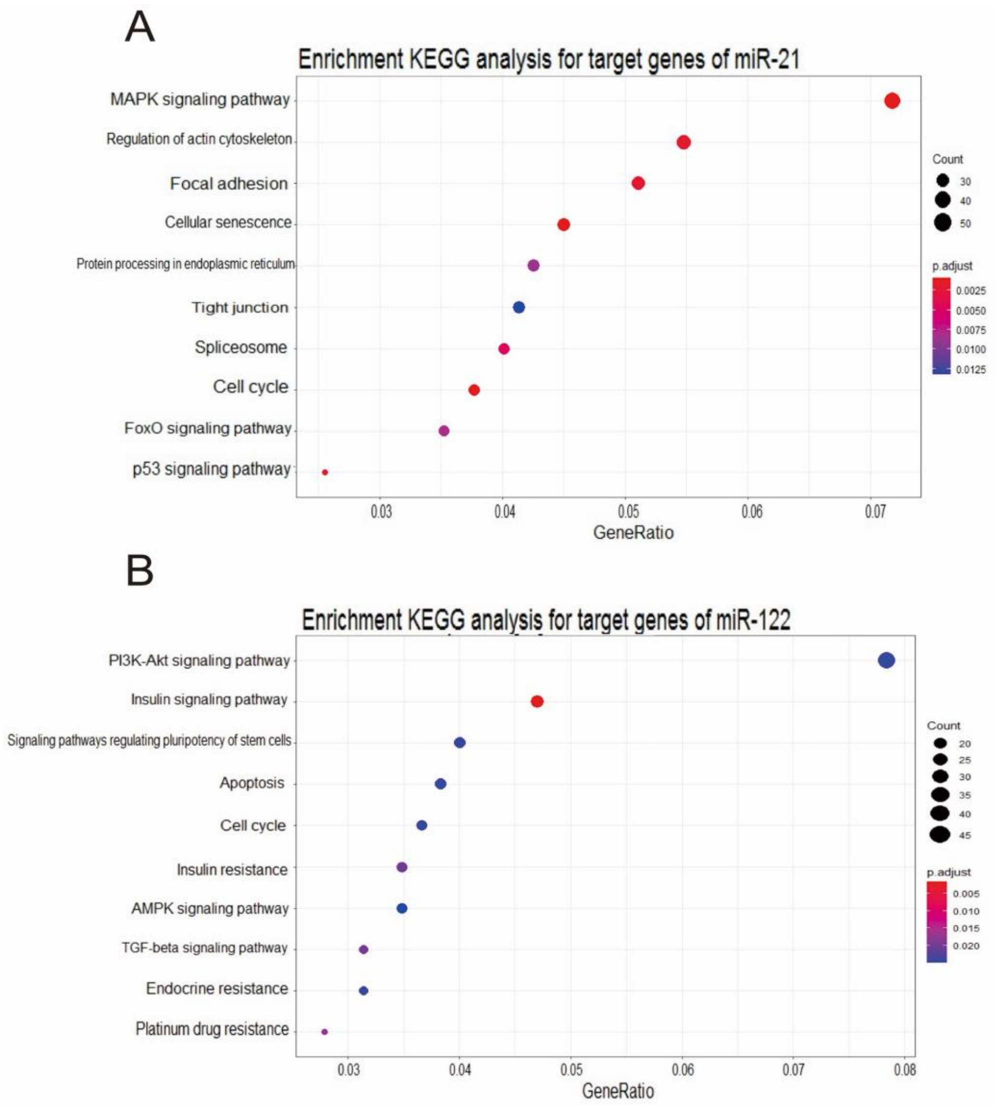

Figure S1. Enrichment analysis for predicted target genes of miR-21 and miR-122. **A-B**, KEGG pathway enrichment analysis for predicted target genes of miR-21 and miR-122 Using Tarbase v7.0, the number of predicted target genes of miR-21, miR-122 was 2181 and 1548, respectively. KEGG pathway analysis revealed that the target genes of miR-21 were extensively involved in iCCA-associated signaling pathways (the MAPK, FoxO and p53 signaling pathway), suggesting the potential roles of miR-21 in iCCA pathogenesis.

Supplementary Tables

Table S1. Baseline characteristics of included participants in the training and validation cohort.

Characteristics	Training cohort (n=668)	Validation cohort (n=333)	*P*
**Age > 60** yrs	269 (40.3%)	146 (43.8%)	0.279
**Male**	369 (55.2%)	199 (59.8%)	0.174
**Diagnosis**			
**iCCA**	230 (34.4%)	129 (38.7%)	
**Control**	438 (65.6%)	204 (61.3%)	
Healthy control	130 (19.5%)	74 (22.2%)	
Benign diseases	196 (29.3%)	47 (14.1%)	
FNH	37 (5.5%)	8 (2.4%)	
Hemangioma	45 (6.7%)	9 (2.7%)	
Cyst	40 (6.0%)	5 (1.5%)	0.181
AML	9 (1.3%)	4 (1.2%)	
Adenoma	14 (2.1%)	5 (1.5%)	
Other benign lesions	51 (7.6%)	16 (4.8%)	
Malignant diseases	112 (16.8%)	83 (24.9%)	
HCC	11 (1.6%)	1 (0.3%)	
CRLM	55 (8.2%)	69 (20.7%)	
Other liver metastases	28 (4.2%)	0 (0.0%)	
Other malignant lesions	18 (2.7%)	13 (3.9%)	

FNH, focal nodular hyperplasia; AML, Angiomyolipoma; HCC, hepatocellular carcinoma; CRLM, colorectal liver metastasis.

Table S2. The diagnostic performance of circulating markers for iCCA in the entire cohort.

Markers	iCCA (n=359) vs. Control (n=642)					*P*
	AUC (95% CI)	NPV (%)	PPV (%)	Sensitivity (%)	Specificity (%)	
**CA19-9**	0.795 (0.769-0.820)	71.1	86.7	62.1	86.8	<0.001
**miR-21**	0.766 (0.739-0.792)	75.6	67.7	55.7	83.8	<0.001
**miR-122**	0.708 (0.678-0.736)	72.5	65.3	64.1	70.4	<0.001
**2-miR model***	0.791 (0.765-0.816)	78.0	70.8	69.1	76.2	<0.001
**3-marker model[&]**	0.855 (0.832-0.876)	82.2	86.0	73.0	87.1	<0.001

* Logit (p = iCCA) = -9.289 + (0.793×miR-21) + (0.353×miR-122) ; [#] Logit (p = iCCA) = -9.967 + 0.777×miR-21 + 0.389×miR-122 + 0.004×CA19-9; AUC, Area Under Curve; CI, confidence interval; NPV, negative predictive value; PPV, positive predictive value.

Table S3. The diagnostic performance of circulating markers between iCCA and healthy control.

markers	iCCA vs. healthy control (n=204)					*P*
	AUC (95% CI)	NPV (%)	PPV (%)	Sensitivity (%)	Specificity (%)	
**CA19-9**	0.838 (0.805-0.867)	63.7	87.2	62.7	96.1	<0.001
**miR-21**	0.834 (0.801-0.864)	72.4	79.9	74.1	79.4	<0.001
**miR-122**	0.698 (0.658-0.735)	44.8	65.7	61.0	75.0	<0.001
**2-miR model***	0.836 (0.803-0.866)	67.6	78.0	73.3	83.8	<0.001
**3-marker model[&]**	0.894 (0.866-0.918)	77.3	86.0	73.5	96.1	<0.001

* Logit (p = iCCA) = -9.289 + (0.793×miR-21) + (0.353×miR-122) ; [#] Logit (p = iCCA) = -9.967 + 0.777×miR-21 + 0.389×miR-122 + 0.004×CA19-9; AUC, Area Under Curve; CI, confidence interval; NPV, negative predictive value; PPV, positive predictive value.

Table S4. The diagnostic performance of circulating markers between iCCA and benign liver lesions.

Markers	iCCA vs. benign liver lesions (n=243)					
	AUC (95% CI)	NPV (%)	PPV (%)	Sensitivity (%)	Specificity (%)	*P*
**CA19-9**	0.810 (0.776-0.840)	63.3	84.2	76.0	75.7	<0.001
**miR-21**	0.729 (0.692-0.764)	64.8	69.7	55.7	76.5	<0.001
**miR-122**	0.728 (0.691-0.763)	59.6	69.1	65.7	71.2	<0.001
**2-miR model***	0.773 (0.737-0.806)	65.9	71.0	61.0	80.7	<0.001
**3-marker model[&]**	0.843 (0.811-0.871)	71.5	77.7	65.7	90.5	<0.001

* Logit (p = iCCA) = -9.289 + (0.793×miR-21) + (0.353×miR-122) ; [#] Logit (p = iCCA) = -9.967 + 0.777×miR-21 + 0.389×miR-122 + 0.004×CA19-9; AUC, Area Under Curve; CI, confidence interval; NPV, negative predictive value; PPV, positive predictive value.

Table S5. The diagnostic performance of circulating markers between iCCA and malignant liver lesions.

Markers	iCCA vs. other malignant liver lesions (n=195)					
	AUC (95% CI)	NPV (%)	PPV (%)	Sensitivity (%)	Specificity (%)	*P*
**CA19-9**	0.733 (0.694-0.769)	100.0	64.8	55.7	84.6	<0.001
**miR-21**	0.742 (0.703-0.778)	61.5	72.2	56.8	80.5	<0.001
**miR-122**	0.693 (0.652-0.731)	51.0	68.4	64.1	68.7	<0.001
**2-miR model***	0.767 (0.730-0.802)	58.0	71.9	47.9	94.4	<0.001
**3-marker model[&]**	0.830 (0.796-0.860)	68.7	77.2	66.0	92.3	<0.001

* Logit (p = iCCA) = -9.289 + (0.793×miR-21) + (0.353×miR-122) ; [#] Logit (p = iCCA) = -9.967 + 0.777×miR-21 + 0.389×miR-122 + 0.004×CA19-9; AUC, Area Under Curve; CI, confidence interval; NPV, negative predictive value; PPV, positive predictive value.

Table S6. The diagnostic performance of circulating markers for iCCA with different AJCC stage.

Markers	iCCA with different AJCC stage vs. Control											
	stage 0-I (n=190) vs. Control						Stage II-IV (n=169) vs. Control					
	AUC (95% CI)	NPV (%)	PPV (%)	Sensitivity (%)	Specificity (%)	*P*	AUC (95% CI)	NPV (%)	PPV (%)	Sensitivity (%)	Specificity (%)	*P*
**CA19-9**	0.779 (0.749-0.806)	80.1	78.6	68.4	75.7	<0.001	0.814 (0.785-0.840)	84.3	85.0	63.3	92.8	<0.001
**miR-21**	0.783 (0.753-0.810)	82.7	81.7	77.9	64.3	<0.001	0.748 (0.716-0.777)	83.8	83.9	52.7	84.3	<0.001
**miR-122**	0.723 (0.691-0.753)	81.2	84.3	63.7	75.4	<0.001	0.690 (0.657-0.722)	81.1	80.8	62.1	70.4	<0.001
**2-miR model***	0.810 (0.782-0.836)	84.4	79.8	64.7	83.6	<0.001	0.770 (0.740-0.99)	84.7	90.0	66.3	76.2	<0.001
**3-marker model[&]**	0.848 (0.821-0.871)	84.9	84.8	70.5	87.2	<0.001	0.864 (0.838-0.887)	87.9	88.2	71.0	93.8	<0.001

* Logit (p = iCCA) = -9.289 + (0.793×miR-21) + (0.353×miR-122) ; [#] Logit (p = iCCA) = -9.967 + 0.777×miR-21 + 0.389×miR-122 + 0.004×CA19-9; AUC, Area Under Curve; CI, confidence interval; NPV, negative predictive value; PPV, positive predictive value.
